# The Study of Performance of a Nanoribbon Biosensor, Sensitized with Aptamers and Antibodies, upon Detection of Core Antigen of Hepatitis C Virus

**DOI:** 10.3390/mi14101946

**Published:** 2023-10-19

**Authors:** Yuri D. Ivanov, Kristina A. Malsagova, Kristina V. Goldaeva, Tatyana O. Pleshakova, Andrey F. Kozlov, Rafael A. Galiullin, Ivan D. Shumov, Vladimir P. Popov, Irina K. Abramova, Vadim S. Ziborov, Oleg F. Petrov, Alexander Yu. Dolgoborodov, Alexander I. Archakov

**Affiliations:** 1 Institute of Biomedical Chemistry (IBMC), 119121 Moscow, Russia; yurii.ivanov.nata@gmail.com (Y.D.I.); kristina.malsagova86@gmail.com (K.A.M.); t.pleshakova1@gmail.com (T.O.P.); afkozlow@mail.ru (A.F.K.); rafael.anvarovich@gmail.com (R.A.G.); shum230988@mail.ru (I.D.S.); ira.abramova.01@inbox.ru (I.K.A.); ziborov.vs@yandex.ru (V.S.Z.); alexander.archakov@ibmc.msk.ru (A.I.A.); 2 Joint Institute for High Temperatures of Russian Academy of Sciences, 125412 Moscow, Russia; ofpetrov@ihed.ras.ru (O.F.P.); aldol@ihed.ras.ru (A.Y.D.); 3 Rzhanov Institute of Semiconductor Physics, Siberian Branch of Russian Academy of Sciences, 630090 Novosibirsk, Russia; popov@isp.nsc.ru

**Keywords:** HCVcoreAg, antibody, aptamer, nanoribbon biosensor, diagnostics

## Abstract

The development of highly sensitive diagnostic systems for the early revelation of diseases in humans is one of the most important tasks of modern biomedical research, and the detection of the core antigen of the hepatitis C virus (HCVcoreAg)—a protein marker of the hepatitis C virus—is just the case. Our study is aimed at testing the performance of the nanoribbon biosensor in the case of the use of two different types of molecular probes: the antibodies and the aptamers against HCVcoreAg. The nanoribbon sensor chips employed are based on “silicon-on-insulator structures” (SOI-NR). Two different HCVcoreAg preparations are tested: recombinant β-galactosidase-conjugated HCVcoreAg (“Virogen”, Watertown, MA, USA) and recombinant HCVcoreAg (“Vector-Best”, Novosibirsk, Russia). Upon the detection of either type of antigen preparation, the lowest concentration of the antigen detectable in buffer with pH 5.1 was found to be approximately equal, amounting to ~10^−15^ M. This value was similar upon the use of either type of molecular probes.

## 1. Introduction

Hepatitis caused by the hepatitis C virus (HCV) represents an urgent problem for public health [1,2]. Globally, more than 170 million people are infected with HCV [3]. This virus has been known since 1989. Despite the tremendous advances achieved in the treatment of this disease over the past decades, many patients may still remain difficult to treat [4,5]. The well-studied structure of HCV core proteins allows one to use them as biomarkers [6,7,8].

HCV core antigen (HCVcoreAg) is a marker protein used for the detection of HCV. This protein is found both in complete HCV virions and in RNA-less core protein structures [9]. The primary sequence of HCVcoreAg is highly conserved among all the different genotypes of the virus [10]. HCVcoreAg appears in blood 10–15 days after infection with HCV (1–2 days later than HCV RNA), thus not hindering early diagnosis of the disease [10,11]. This is why HCVcoreAg represents a promising protein marker for the early revelation of HCV. Worldwide, a wide variety of different analytical systems for the detection of HCVcoreAg have been proposed [12,13,14].

To date, enzyme-linked immunosorbent assay (ELISA)-based methods of HCVcoreAg detection are routinely used in clinical practice [15]. Once again, it is the early diagnosis of HCV infection for which the use of HCVcoreAg as a marker is promising for, and the most serious drawback of ELISA-based assays is the lack of sensitivity [16,17]. With regard to early diagnosis of diseases, methods with at least femtomolar (or, better, subfemtomolar, <10^−15^ M) detection limits are required [17]. Rissin et al. [17] emphasized that at the earliest stages of socially significant viral infectious diseases, such as the human immunodeficiency virus (HIV) infection, the serum levels of their protein markers range from 10^−17^ M to 10^−14^ M. This indicates the clinical importance of the subfemtomolar concentration range of protein biomarkers of viral infections. In this regard, nanotechnology-based methods open the way to overcome this detection limit threshold [18]. Among these methods, nanowire and nanoribbon-based systems should be singled out [18]. These systems have numerous advantages. The first one is the label-free detection [19]. Furthermore, the presence of multiple sensing elements (i.e., an array of nanowires or nanoribbons) on a single sensor chip provides multiplexed detection [20]. The third advantage is rapid analysis. Namely, these biosensors allow one to detect the target analyte within ~15–20 min in a small sample volume [19]. Owing to these advantages, this type of biosensor has found its applications in highly sensitive real-time detection of a number of various types of analytes (such as nucleic acids, proteins, and viral particles) at low and ultra-low (femto- and subfemtomolar) concentrations, as was demonstrated in numerous papers [21,22,23,24,25,26,27,28,29,30,31]. This explains why this type of biosensor is so attractive for use in the early revelation of various diseases in humans.

In our present research, we have employed the nanoribbon biosensor-based approach to the detection of HCVcoreAg in order to compare the biosensor performance upon the use of different molecular probes for the capturing of the target protein. Namely, either anti-HCVcoreAg aptamers or anti-HCVcoreAg antibodies have been employed in our biosensor experiments as nanoribbon-immobilized molecular probes against the target HCVcoreAg protein. Furthermore, two model samples of purified HCVcoreAg preparation, which were manufactured by either “Virogen” (Watertown, MA, USA) or “Vector-Best” (Russia), have been studied. In the biosensor, sensor chips based on the so-called “silicon-on-insulator” structures have been employed. These chips have been fabricated by photolithography and gas-phase etching. We have demonstrated that these sensor chips, whose surface has been sensitized with either antibodies or anti-HCVcoreAg aptamers, can be successfully employed for label-free real-time detection of HCVcoreAg manufactured by “Virogen” or “Vector-Best” with virtually equal efficiency.

## 2. Materials and Methods

### 2.1. Reagents

The following reagents were used to pre-clean the sensing surface of the SOI-NR chip and perform its chemical modification: hydrofluoric acid (HF), 96% ethanol (C_2_H_5_OH) (“Reakhim”, Moscow, Russia), isopropanol, purified to 99.9% (C_3_H_8_O) (“Acros Organics”, Geel, Belgium), 3-aminopropyltriethoxysilane (APTES) (“Sigma Aldrich”, St.-Louis, MO, USA) [29].

The sensitization of the nanoribbon surface with molecular probes was performed by their covalent immobilization onto the surface of individual nanoribbons using 3,3′-dithiobis (sulfosuccinimidyl propionate (DTSSP) (Thermo Scientific, Waltham, MA, USA) as a crosslinker.

The following auxiliary chemicals were also used in this study: potassium phosphate buffer (PPB) and potassium phthalate buffer (PPhthB).

Deionized water was purified using a Millipore Simplicity UV water purification system (“Millipore”, Molsheim, France).

### 2.2. Proteins

#### 2.2.1. D-NFATc1

D-NFATc1 represents a domain of NFATc1 globular protein, whose isoelectric point is 4.7 according to theoretical calculations performed with ExPASy: SIB Bioinformatics Resource Portal. The preparation of this protein was kindly provided by Dr. A.Yu. Rubina (Engelhardt Institute of Molecular Biology, RAS, Moscow, Russia).

#### 2.2.2. Anti-HCVcoreAg Antibodies

Murine monoclonal anti-HCVcoreAg antibodies (clone 1E5, specificity: the 1–80 a.a.r. region of the HCV core protein) purchased from “Virogen” (Watertown, MA, USA) were used.

The antibodies were specific to anti-HCVcoreAg antigen employed as a target.

### 2.3. Aptamers against HCVcoreAg

Anti-HCVcoreAg aptamers procured from “Evrogen” JSC (Moscow, Russia) with the sequence 5′-NH_2_-(T)_10_-ACGCTCGGATGCCACTACAGGCACGCCAGACCAGCCGTCCTCTCTTCATCCGAGCCTTCACCGAGCCTCATGGACGTGCTGGTGA-3′ [32] were employed.

### 2.4. Antigens

The following antigens were employed in this study: (1) recombinant hepatitis C virus core protein (HCVcoreAg, 22 kDa) modified at the N-terminus with β-galactosidase (114 kDa) (“Virogen”, Watertown, MA, USA), pI = 8.9; (2) recombinant hepatitis C virus core protein HCVcoreAg (kindly provided by O.N. Yastrebova, “Vector-Best”, Russia).

### 2.5. Preparing Buffer Solutions of HCVcoreAg

HCVcoreAg solutions with concentrations ranging from 10^−15^ M to 10^−13^ M were prepared from the initial protein solution (2 μM in 50 mM PPB, pH 7.4) by tenfold serial dilution with 1 mM PPhthB (pH 5.1).

Each solution prepared was incubated in the shaker at 10 °C for 30 min. Protein solutions were prepared immediately prior to the measurements.

### 2.6. The Sensor Chips

**Figure 1:**
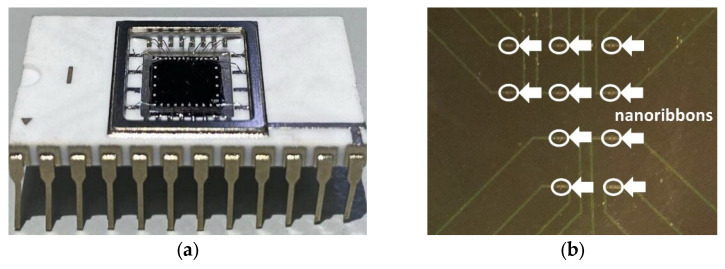
(**a**) A photographic image of the SOI-NR sensor chip. (**b**) Optical image of the sensitive area of the chip. White arrows indicate the nanoribbons. The diameter of the sensitive area of the chip is ~2 mm.

The sensor chip is based on an ultrasensitive field-effect nanotransistor, in which the nanoribbon surface acts as a virtual gate [29,33]. The sensor chip was fabricated on the basis of “silicon-on-insulator” (SOI) structures. In general, the fabrication technology was analogous to the Smart Cut one but with a number of differences. First, buried oxide (BOX) was not subjected to hydrogen implantation. Second, the interface between BOX and upper layer of silicon represented a bonded surface. Such an approach allowed us to minimize the risk of occurrence of defects in Si/SiO_2_ system, providing stability of parameters of the SOI structures [33]. The total initial thickness of the SOI layers was 500 nm. It was subsequently decreased in the course of numerous steps of thermal oxidation. As a result, the thickness of the BOX layer and that of the cut-off layer became 300 nm and 30 nm, respectively. The SOI samples were structured by means of optical lithography in order to form nanoribbon structures located between contact pads. The ohmic contacts were formed by deposition of a polysilicon layer with subsequent doping. In order to provide lateral structuring of the SOI layers, lithography and gas plasma-chemical etching were employed. In [33], the formation of SOI layers is described in more detail. A photographic image of the SOI-NR chip employed in our biosensor, and optical image of the sensitive area of the chip are shown in Figure 1.

**Figure 2:**
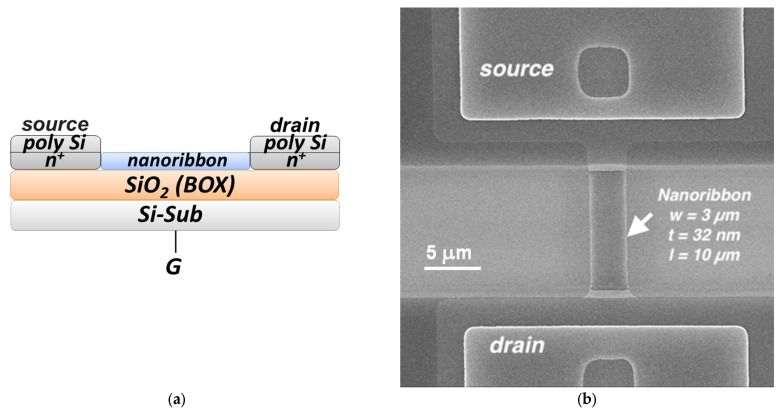
Schematic drawing of the cross-section of an individual nanoribbon based on the silicon-on-insulator structures (**a**) and scanning electron microscopy (SEM) image of an individual nanoribbon (**b**). The white arrow shows the nanoribbon position between source and drain electrodes. Nanoribbon dimensions: thickness *t* = 32 nm, width *w* = 3 µm, length *l* = 10 µm.

The chips comprised five pairs of nanoribbons. The nanoribbons were 3 µm wide, 32 nm thick, and 10 µm long [31]. The nanoribbon structures had n-type conductivity. Figure 2 displays a schematic drawing of the cross-section of an individual nanoribbon and a scanning electron microscopy (SEM) image of an individual nanoribbon.

### 2.7. Modification of the Surface of the Sensor Chip

Chemical modification of the sensing surface involved its pretreatment and further silanization with 3-aminopropyltriethoxysilane (APTES) analogously to the method developed by Yamada et al. [34] as described elsewhere [29,35].

At the pretreatment stage, mechanical contaminants were removed from the chip surface by rinsing them with isopropanol (C_3_H_7_OH). A solution containing hydrofluoric acid (HF) and ethanol (C_2_H_5_OH) was further applied in order to remove any resting organic contaminants and, subsequently, the natural oxide [35,36], which was formed during storage of the chip [36].

In order to form hydroxyl groups on the nanoribbons’ surface, the sensor chip was treated in a UV Ozone Cleaner (ProCleaner™ Plus, Ossila Ltd., Sheffield, UK) for one hour. Vapor-phase silanization of the chip surface with APTES [34] was then carried out [29].

### 2.8. Sensitization of Individual Nanoribbons

The surface of individual nanoribbons was sensitized with molecular probes against the target HCVcoreAg molecules in order to provide their biospecific detection. In other words, agents increasing the likelihood of selective binding between the target molecules and the nanoribbon surface are applied to it. These agents also provide high detection sensitivity [37].

The nanoribbon sensitization was performed by covalent immobilization of molecular probes of either type onto the surface of individual nanoribbons after the silanization of the sensor chip surface. DTSSP was used as a crosslinker for the immobilization [31]. The freshly prepared DTSSP crosslinker solution was placed into a shaker and kept there at 10 °C for 10 min. The crosslinker solution was then dispensed onto the sensor chip surface and incubated thereon at 15 °C and 80% humidity for 30 min. The chip surface was then rinsed ten times with 1 mL of deionized water. After that, 1 µM solutions of either anti-HCVcoreAg antibodies or anti-HCVcoreAg aptamers were applied onto the surface of individual nanoribbons using a Piezorray dispensing system (PerkinElmer, Inc., Waltham, MA, USA). The dispensed volume was ~3 nL. In order to remove any unbound molecular probes from the surface of the nanoribbons, the sensor chip was washed with 50 mL of warm deionized water.

The nanoribbons with immobilized antibody or aptamer molecular probes were used as working nanoribbons, while nanoribbons without immobilized molecular probes were used as control ones.

### 2.9. The Biosensor Setup

**Figure 3:**
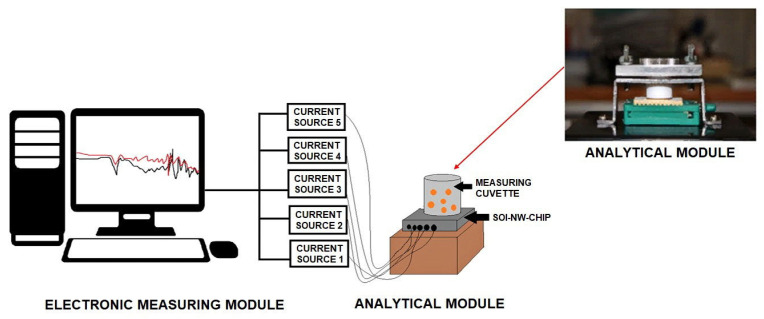
Schematic of the nanoribbon biosensor, which comprised of the analytical and the electronic measuring modules.

The nanoribbon biosensor represents a system consisting of two modules: the analytical one and the electronic measuring one (Figure 3).

The analytical module was used for all operations involving liquids, including the solutions of the target protein. The interaction of the target protein molecules with the chip surface occurred within this module. The analytical module included a measuring cell of 1000 µL capacity. The bottom of the cell was formed by the sensor chip. A stirrer was employed for the enhancement of the delivery of target protein molecules to the sensor chip surface. The stirring rate was 1000 rpm.

The electronic measuring module was used for the treatment and processing of the signal received from the sensor chip. The signal from the nanoribbons was received and recorded in real-time by the electronic measuring module. This module allowed us to simultaneously receive signals from all nanoribbons of one and the same sensor chip and visualize the obtained data on a PC monitor during the experiment in real-time. Analog-to-digital conversion of the recorded signal, as well as analysis and visualization of the measurement results, was performed using the TURBO NBS software (Rospatent registration No. 2015612969, 27 February 2015). The so-obtained data were stored in the form of working file. The electronic measuring module allowed us to record current–voltage characteristics (CVCs) of the nanoribbon sensors and measurements and to perform real-time measurements by recording the time dependencies of the drain–source current *I_ds_*.

### 2.10. Electrical Measurements

Electrical measurements were conducted using a Keithley picoammeter (Model 6487, Keithley Instruments Inc., Cleveland, OH, USA). A substrate of the SOI structures was used as a gate during the measurements.

The nanoribbon biosensor system allows one to measure electrical signals in two modes:

1. The real-time mode: measuring *I_ds_(t)* (recording drain–source current *(I_ds_)* vs. experiment duration *(t)*);
2. The mode of recording the CVCs of the nanoribbons: measuring *I_ds_(V_g_)* (recording drain–source current *(I_ds_)* vs. applied voltage *(V_g_)*).

**Figure 4:**
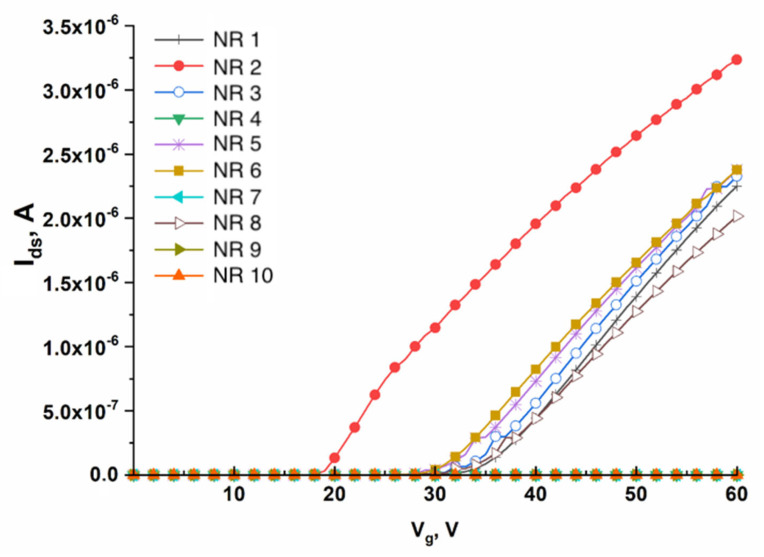
Typical CVC curves recorded for 10 nanoribbons arranged on a single SOI-NR sensor chip. Experimental conditions: gate voltage *V_g_* = 0 ÷ 60 V, *V_ds_* = 0.1 V, PPhthB buffer (pH 5.1).

The working value of gate voltage (the working point) *V_g_*, at which the drain–source current *(I_ds_)* was recorded, had been selected prior to performing the biosensor measurements. The working point was determined by measuring the current–voltage characteristics in a buffer solution. The *I_ds_(V_g_)* dependence was recorded within the voltage range from 0 to 60 V. Figure 4 displays typical CVC curves obtained in this way.

In order to avoid the problems related to Debye screening, the salt concentration in the buffer solutions used in the biosensor experiments on the detection target protein was low (1 mM). At this concentration of buffer salts, the Debye length is ~12 nm, which is sufficient for detecting signals induced by HCVcoreAg binding to the nanoribbon surface [38,39].

Measurements were carried out at a constant voltage between the ohmic contacts of the nanoribbons (source–drain) *V_ds_* = 0.1 V and at operating gate voltage *V_g_* = 45 V. Once the measurement system was switched on, the cell was filled with the working solution and sequential measurements were then carried out: at the initial time interval before the test sample had been added; after the test solution had been added to the cell; and when the test solution in the cell was replaced with pure washing buffer.

### 2.11. Detection of HCVcoreAg with the Nanoribbon Biosensor

**Figure 5:**
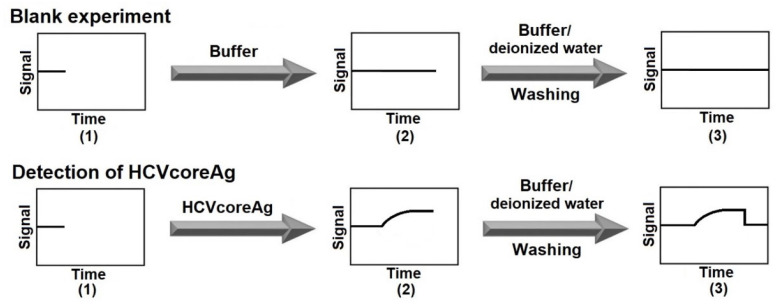
Schematic of the experiment workflow upon the use of SOI-NR sensor chip of n-type conductance. Numbers indicate the main steps of the experiment: recording the baseline in pure protein-free buffer PPhthB, pH 5.1 (1); recording the signal after the addition of pure protein-free buffer (in the blank experiment) or the HCVcoreAg solution (upon the target protein detection) (2); and wash with pure protein-free buffer and warm deionized water (3).

Figure 5 displays a schematic of the experiment workflow upon the use of SOI-NR sensor chip of n-type conductance.

A sensor chip, whose individual nanoribbons were sensitized with either anti-HCVcoreAg antibodies or anti-HCVcoreAg aptamers, as described above in Section 2.8, was used in this study. Prior to the measurement, pure PPhthB buffer was pipetted into the measuring cell in order to record the baseline. In order to detect HCVcoreAg in purified buffer solution, 150 µL of buffer solution of HCVcoreAg at concentration ranging from 10^−16^ M to 10^−13^ M was pipetted into the measuring cell containing 300 µL of 1 mM buffer solution (PPhthB, pH 5.1). The measurements were conducted starting with the lowest concentration. After each test run, the sensor chip surface was washed with pure protein-free buffer (PPhthB, pH 5.1) and then with warm deionized water (50 mL).

In the blank experiments, pure protein-free buffer (PPhthB, pH 5.1) was pipetted into the measuring cell instead of the HCVcoreAg solution. The sensor chip surface was washed with the same buffer, followed by washing with warm deionized water (50 mL).

### 2.12. Data Analysis

The data obtained in the real-time mode were presented as sensorgrams, which displayed time dependence of a dimensionless parameter expressed as arbitrary units.

Changes in the current *I_ds_*, recorded for each nanoribbon, were first normalized to unity by dividing their values by the initial current. Next, in order to take into account the non-specific interactions, the values obtained in the blank experiment were subtracted from the absolute data obtained with HCVcoreAg solution. The differential signal was then calculated by subtracting the signal for the control nanoribbon from the signal for the working nanoribbon. The resulting time dependencies of the current *I_ds_(t)* were presented as sensorgrams showing the differential signal.

This approach to data processing has allowed us to account for the fact that the initial characteristics of currents flowing through different nanoribbons on one and the same sensor chip can differ. The initial currents for different nanoribbons may differ by one or two orders of magnitude. The standard deviation function was used to confirm the validity of the results. On the sensorgrams, the standard deviation is indicated by error bars.

## 3. Results

### 3.1. Detection of HCVcoreAg with the Use of Antibody Molecular Probes

**Figure 6:**
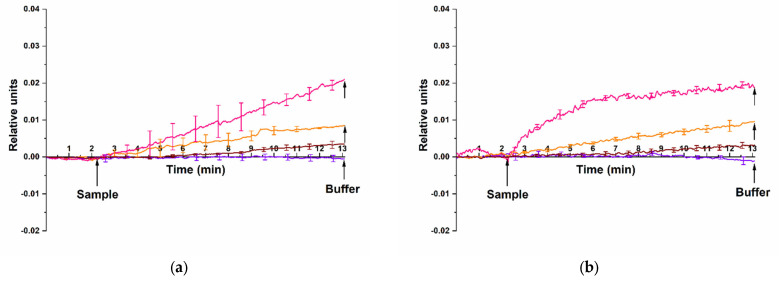
Typical sensorgrams illustrating the detection of HCVcoreAg with a nanoribbon biosensor sensitized with antibody probes. Two different HCVcoreAg preparations were tested: (**a**) HCVcoreAg manufactured by “Virogen”; (**b**) HCVcoreAg manufactured by “Vector-Best”. The HCVcoreAg concentration was 10^−15^ M (brown curve), 10^−14^ M (orange curve) or 10^−13^ M (pink curve). The data obtained in blank experiments for pure PPhthB containing no protein molecules are shown with a violet curve. The SOI-NR chip had n-type conductivity. Experimental conditions: 1 mM PPhthB; *V_g_* = 45 V, *V_ds_* = 0.1 V; solution volume in the measuring cell was 450 µL. All measurements were performed in three technical replicates, and the respective standard deviation is indicated by error bars. Arrows indicate the time points of pipetting the HCVcoreAg solution into the cell and of sensor chip washing with pure buffer.

We have performed the experiments on the HCVcoreAg detection with nanoribbons sensitized with anti-HCVcoreAg antibody probes. Two different HCVcoreAg preparations, produced by two different manufacturers, “Virogen” (Watertown, MA, USA) and “Vector-Best” (Russia) have been tested. Figure 6 displays typical sensorgrams obtained in the course of detection of HCVcoreAg at concentrations ranging from 10^−15^ M to 10^−13^ M in 1 mM PPhthB buffer (pH 5.1). It is to be emphasized that no signal was detected at the lowest (10^−16^ M) HCVcoreAg concentration tested.

The sensorgrams shown in Figure 6 clearly indicate that the addition of HCVcoreAg protein solutions manufactured by either “Virogen” (Figure 6a, brown, orange, and pink curves) or “Vector-Best” (Figure 6b, brown, orange, and pink curves) at concentrations ranging from 10^−15^ M to 10^−13^ M induced an increase in the conductivity of the nanoribbons sensitized with anti-HCVcoreAg antibody probes. This was caused by the positive charge of the target HCVcoreAg protein molecules under the experimental conditions at an acidic pH of 5.1. Accordingly, their binding to the probes immobilized on the surface of silicon nanoribbon altered its conductivity. We also observed an expected decrease in the level of the recorded signal upon decreasing the concentration of the target protein from 10^−13^ M to 10^−15^ M. It is to be emphasized that upon the use of HCVcoreAg preparation from “Virogen”, the signal recorded at 10^−13^ M increased continuously (Figure 6a, pink curve). Upon the use of HCVcoreAg preparation from “Vector-Best” at the same concentration (10^−13^ M), the signal tended to reach a plateau value (Figure 6b, pink curve). This difference in the sensorgram shape can be caused by the modification of HCVcoreAg from “Virogen” with β-galactosidase at the N-terminus, while HCVcoreAg from “Vector-Best” was devoid of such a modification. The β-galactosidase modification can change the target protein conformation and, hence, the binding affinity and the stability upon its interaction with nanoribbon-immobilized molecular probes. Furthermore, the analysis of the blank solution revealed no change in conductivity of the antibody-sensitized nanoribbons (Figure 6a,b, violet curve), thus indicating that the biosensor does not respond to non-specific interactions or other interferences from the buffer solution components.

The data obtained in our experiments indicated that recombinant HCVcoreAg manufactured by both “Virogen” and “Vector-Best” was detected using antibody molecular probes with comparable sensitivity. At that, the lowest concentration of HCVcoreAg detectable in buffer solution with pH 5.1 was determined to be 10^−15^ M.

### 3.2. Detection of HCVcoreAg with the Use of Aptamer Molecular Probes

In the tests performed at this stage, similar to the experiments described above, two different HCVcoreAg preparations from “Virogen” (USA) and “Vector-Best” (Russia) were used. However, the nanoribbons were sensitized with covalently immobilized aptamers instead of antibodies.

**Figure 7:**
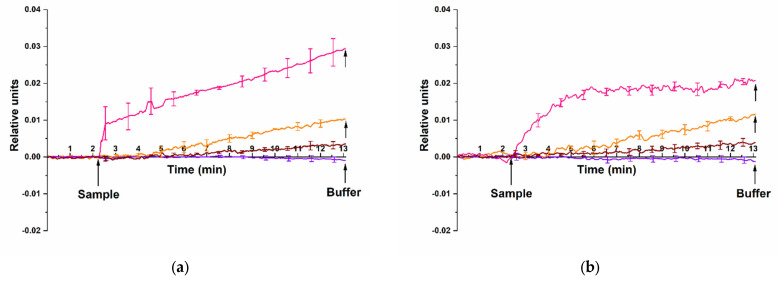
Typical sensorgrams illustrating the detection of HCVcoreAg with a nanoribbon biosensor sensitized with aptamer probes. Two different HCVcoreAg preparations were tested: (**a**) HCVcoreAg manufactured by “Virogen”; (**b**) HCVcoreAg manufactured by “Vector-Best”. The HCVcoreAg concentration was 10^−15^ M (brown curve), 10^−14^ M (orange curve) or 10^−13^ M (pink curve). The data obtained in blank experiments for pure PPhthB containing no protein molecules are shown with a violet curve. The SOI-NR chip had n-type conductivity. Experimental conditions: 1 mM PPhthB; *V_g_* = 45 V, *V_ds_* = 0.1 V; solution volume in the measuring cell was 450 µL. All measurements were performed in three technical replicates, and the respective standard deviation is indicated by error bars. Arrows indicate the time points of pipetting the HCVcoreAg solution into the cell and of sensor chip washing with pure buffer.

It was found that both antigen samples can be successfully detected in the solution using nanoribbons sensitized with aptamer probes. Figure 7 displays typical sensorgrams obtained in the course of detection of HCVcoreAg in the solution at concentrations ranging from 10^−15^ M to 10^−13^ M using n-type nanoribbons with immobilized aptamer probes. Upon the analysis of 10^−16^ M HCVcoreAg solution, no signal was recorded upon the addition of the analyzed solution into the measuring cell.

The sensorgrams shown in Figure 7 indicate that upon addition of HCVcoreAg solutions at concentrations ranging from 10^−15^ M to 10^−13^ M, the nanoribbon conductivity expectedly increases due to binding of the positively charged HCVcoreAg antigen molecules with the aptamer-sensitized nanoribbon surface. This was observed for both HCVcoreAg preparations tested, which were purchased from either “Virogen” (Figure 7a, brown, orange, and pink curves) or “Vector-Best” (Figure 7b, brown, orange, and pink curves). It should be emphasized that, similar to the case with antibodies as molecular probes, the dynamic response at 10^−13^ M HCVcoreAg concentration differs upon the use of different preparations of the target protein. Namely, upon the analysis of HCVcoreAg preparation from “Virogen” (Figure 7a, pink curve) and of that from “Vector-Best” (Figure 7b, pink curve), the shape of the recorded sensorgram curves differs. This can be explained by the presence of β-galactosidase fragment at the N-terminus of HCVcoreAg from “Virogen”, which influences the interaction of the target protein with nanoribbon-immobilized molecular probes. In contrast, the analysis of the blank solution revealed no change in the conductivity of aptamer-sensitized nanoribbons (Figure 7a,b, violet curve), demonstrating no response to non-specific interactions or interferences from the buffer components.

Thus, in our experiments, the lowest concentration of the target protein (recombinant HCVcoreAg), detectable with the use of aptamer molecular probes, amounted to 10^−15^ M, being equal upon the analysis of either of the two different HCVcoreAg preparations (from “Virogen” or from “Vector-Best”).

### 3.3. Analysis Selectivity Check

In order to check the selectivity of the analysis performed with the nanoribbon biosensor, additional experiments with the use of a non-specific protein (D-NFATc1) instead of the target protein (HCVcoreAg) were performed. The experiments with the non-specific protein were performed at the highest (10^−13^ M) concentration studied. For either type of molecular probe, no change in the biosensor signal was registered after the addition of the D-NFATc1 protein.

## 4. Discussion

Nanoribbon biosensor is a unique platform for high-sensitivity detection of protein molecules. It allows one to register a signal, which corresponds to a level of a single molecule per the nanoribbon sensing element [40] since the latter has quite a high surface-to-volume ratio [41].

Currently, ELISA and polymerase chain reaction (PCR) are the key methods used for diagnosing HCV [42,43,44]. One of the significant drawbacks of PCR is that data interpretation can be impeded (in particular, for the samples with viral loads below the quantitation limit) [45]. Moreover, PCR is quite sensitive to sample contamination—as opposed to the methods based on the detection of protein markers. Furthermore, relatively expensive equipment and reagents are required for PCR-based tests [46]. In turn, ELISA may yield false negative results in immunocompromised patients [47]. Furthermore, this analysis can yield low positive prognostic values in cohorts with low (<10%) prevalence of HCV infection [48,49]. In addition, as was noted in the Introduction, ELISA allows one to detect biomarkers with a concentration sensitivity of ~10^−12^ M [17], which is insufficient for diagnosing asymptomatic hepatitis C [17].

It is promising to use a nanoribbon biosensor as a universal platform for large-scale manufacturing of highly sensitive diagnostic systems available for personalized use owing to the ability of this biosensor to detect protein markers of infectious diseases with high sensitivity [50,51].

Our data reported herein indicate that both anti-HCVcoreAg aptamer probes and anti-HCVcoreAg antibody probes immobilized on the nanoribbon surface have allowed us to successfully detect the target HCVcoreAg protein in buffer solutions at ultra-low concentrations (10^−15^ M). The conductivity of n-type nanoribbons expectedly increased upon the pipetting of the HCVcoreAg solution into the measuring cell.

Hence, the detection sensitivity upon the use of aptamer molecular probes has been found to be comparable with that attained upon the use of antibodies. However, aptamer probes have a number of advantages over antibodies—namely, higher chemical, temperature, time stability, and low production cost. Accordingly, aptamers are preferred to be used as molecular probes [52].

Apart from the size of the molecular probes (such as aptamers and antibodies), the change in the signal observed can also be influenced by other factors, such as steric hindrances and the accessibility of the binding site. The relative efficiency of the aptamers and the antibodies as molecular probes depends on specific binding interactions and on the analysis conditions. Aptamers are shorter, and steric hindrances may thus affect their binding with the target. This may occur when the accessibility of the nanoribbon surface is hindered as a result of a binding event. Moreover, if the aptamer probe does not have optimal orientation/conformation upon the binding, the latter may not cause any significant change in the biosensor signal. It should be noted that our study is just aimed at the demonstration of the capabilities of our biosensor upon the use of aptamers and antibodies as molecular probes for the detection of HCVcoreAg—though the title of our manuscript may imply a direct comparison. With both types of molecular probes, we have successfully demonstrated the reliable detection of HCVcoreAg at ultra-low concentrations, thus emphasizing the biosensor’s versatility. Actually, our study is aimed at confirming the fact that both the aptamer and the antibody molecular probes can be successfully employed for the detection of HCVcoreAg with the nanoribbon biosensor. We hope that our results reported can be used as a starting point for future research aimed at studying comparative aspects of these types of molecular probes in detail.

The detection sensitivity has been found to be comparable upon the use of either of the HCVcoreAg preparations produced by “Virogen” and “Vector-Best”. It is also worth mentioning that the HCVcoreAg preparation produced by “Virogen” represents a conjugate of the target protein with β-galactosidase. This conjugate, accordingly, has a higher molecular weight than the protein in the preparation manufactured by “Vector-Best”. It is to be emphasized that under the conditions of our experiments, this fact has not affected the detection sensitivity. Furthermore, analysis of the blank solutions containing no HCVcoreAg has revealed no changes in the nanoribbon conductivity. At that, the addition of non-specific D-NFATc1 protein at high (10^−13^ M) concentration has not led to any change in the biosensor signal, thus confirming the selectivity of the analysis performed. It is to be emphasized that though the detection limit was the same upon the use of different HCVcoreAg preparations, the shape of the kinetic curves recorded was different for the β-galactosidase-modified HCVcoreAg from Virogen—in comparison with HCVcoreAg from VectorBest (which was devoid of such a modification). This phenomenon indicates that it is very important to consider the possible influence of various modifications on the protein binding efficiency. Potential differences in the binding efficiency of antigen preparations from different manufacturers must be tested in order to estimate the reliability of their use in analytical systems intended for the early diagnosis of diseases in humans.

## 5. Conclusions

In our biosensor experiments on the detection of HCVcoreAg protein with a nanoribbon biosensor, the nanoribbons were sensitized with covalently immobilized molecular probes of two types: antibodies and aptamers against HCVcoreAg. Furthermore, two different HCVcoreAg preparations have been tested. The first one was the β-galactosidase conjugate of recombinant HCVcoreAg produced by “Virogen” (Watertown, MA, USA). The second one was recombinant hepatitis C virus core protein HCVcoreAg (produced by “Vector-Best”, Russia). The measurements have been carried out in real-time mode for ~15 min. Upon the use of either type of antigen preparation in the experiments performed in purified buffer at pH 5.1, the lowest detectable concentration of the antigen was found to be approximately equal, amounting to ~10^−15^ M. This value was similar upon the use of either aptamer or antibody molecular probes. The biosensor used in our present study is a prototype of a unique test kit, which represents a molecular detector opening up new avenues for the detection of target molecules in solutions at low and ultra-low concentrations. We believe that this biosensor will find its application in the early revelation of serological protein markers of socially significant diseases in humans, thus reducing the mortality rate, as well as improving drug therapy effectiveness and patients’ quality of life.

## Data Availability

The data obtained throughout the experiments can be provided by Yu.D.I. upon reasonable request.
